# Broadening the Berlin definition of ARDS to patients receiving high-flow nasal oxygen: an observational study in patients with acute hypoxemic respiratory failure due to COVID-19

**DOI:** 10.1186/s13613-023-01161-6

**Published:** 2023-07-14

**Authors:** Fleur-Stefanie L. I. M. van der Ven, Christel M. A. Valk, Siebe Blok, Michelle G. Brouwer, Dai Ming Go, Amanda Lokhorst, Pien Swart, David M. P. van Meenen, Frederique Paulus, Marcus J. Schultz, for the PRoAcT–COVID study investigators, E Aydeniz, P van de Berg, D C Bergmans, M Bevers, S den Boer, L S Boers, L D Bos, M Botta, L A Buiteman-Kruizinga, W Coene, M Delmte, Vincenzo Di Leo, D A Dongelmans, T P Dormans, L M Elting, A A Esmeijer, M G de Abreu, A R Girbes, M J de Graaff, D M Go, R L Goossen, H J Hansen, J J Haringman, L Hol, M W Hollmann, P L van der Heiden, J Horn, L E van Ingen, N P Juffermans, M A Kuiper, L J Kuipers, E Koornstra, A Lokhorst, S G Nijbroek, I Martin-Loeches, D M van Meenen, G Mazzinari, S Myatra, F Paulus, M Offermans, T Pisters, A Prins, P van Oosten, J Pillay, I M Purmer, A S Rezaee, T C D Rettig, O Roca, N M Rosenberg, N Schavemaker, A A Sciascera, M J Schultz, A Serpa Neto, G Shrestha, M E Sleeswijk, W Stilma, A C Strang, A C Spronk, P R Tuinman, A M Tsonas, C M A Valk, M Verboom, A P Vlaar, W H van der Ven, P van Velzen, E J Verhoef, T D Vermeulen, P van Vliet, J J Voorham, P H van der Voort, M van der Woude, Weiner, N Yaali, J M Zandvliet, A R van Zanten, T Z van Zijl, S A Zonneveld

**Affiliations:** 1 Department of Intensive Care, Amsterdam UMC, Location AMC,, Amsterdam, The Netherlands; 2 Department of Anesthesiology, Amsterdam UMC, Location AMC, Amsterdam, The Netherlands; 3 Laboratory of Experimental Intensive Care and Anesthesiology (L.E.I.C.A), Amsterdam UMC, Location AMC, Amsterdam, The Netherlands; 4 Center of Expertise Urban Vitality, Faculty of Health, Amsterdam University of Applied Sciences, Amsterdam, The Netherlands; 5 Mahidol–Oxford Tropical Medicine Research Unit (MORU), Mahidol University, Bangkok, Thailand; 6 Nuffield Department of Medicine, University of Oxford, Oxford, UK; 7 Department of Intensive Care, Rode Kruis Ziekenhuis, Beverwijk, The Netherlands

## Abstract

**Background:**

High-flow nasal oxygen (HFNO) is increasingly used in patients with acute hypoxemic respiratory failure. It is uncertain whether a broadened Berlin definition of acute respiratory distress syndrome (ARDS), in which ARDS can be diagnosed in patients who are not receiving ventilation, results in similar groups of patients receiving HFNO as in patients receiving ventilation.

**Methods:**

We applied a broadened definition of ARDS in a multicenter, observational study in adult critically ill patients with acute hypoxemic respiratory failure due to coronavirus disease 2019 (COVID-19), wherein the requirement for a minimal level of 5 cm H_2_O PEEP with ventilation is replaced by a minimal level of airflow rate with HFNO, and compared baseline characteristics and outcomes between patients receiving HFNO and patients receiving ventilation. The primary endpoint was ICU mortality. We also compared outcomes in risk for death groups using the PaO_2_/FiO_2_ cutoffs as used successfully in the original definition of ARDS. Secondary endpoints were hospital mortality; mortality on days 28 and 90; need for ventilation within 7 days in patients that started with HFNO; the number of days free from HFNO or ventilation; and ICU and hospital length of stay.

**Results:**

Of 728 included patients, 229 patients started with HFNO and 499 patients with ventilation. All patients fulfilled the broadened Berlin definition of ARDS. Patients receiving HFNO had lower disease severity scores and lower PaO_2_/FiO_2_ than patients receiving ventilation. ICU mortality was lower in receiving HFNO (22.7 vs 35.6%; *p* = 0.001). Using PaO_2_/FiO_2_ cutoffs for mild, moderate and severe arterial hypoxemia created groups with an ICU mortality of 16.7%, 22.0%, and 23.5% (*p* = 0.906) versus 19.1%, 37.9% and 41.4% (*p* = 0.002), in patients receiving HFNO versus patients receiving ventilation, respectively.

**Conclusions:**

Using a broadened definition of ARDS may facilitate an earlier diagnosis of ARDS in patients receiving HFNO; however, ARDS patients receiving HFNO and ARDS patients receiving ventilation have distinct baseline characteristics and mortality rates.

*Trial registration*: The study is registered at ClinicalTrials.gov (identifier NCT04719182).

**Supplementary Information:**

The online version contains supplementary material available at 10.1186/s13613-023-01161-6.

## Background

The Berlin definition of acute respiratory distress syndrome (ARDS) provides validated support for three levels of arterial hypoxemia that correlate well with mortality in critically ill patients with acute hypoxemic respiratory failure receiving non-invasive ventilation (NIV) or invasive ventilation [1, 2]. High-flow nasal oxygen (HFNO) is increasingly used in patients with acute hypoxemic respiratory failure [3–5], and it is likely that these patients meet the criteria for ARDS if they would receive ventilation [6–8]. The prerequisite of a minimal level of positive end-expiratory pressure (PEEP) in the current definition prevents its use in patients receiving HFNO [9].

Recently, it was suggested to broaden the Berlin definition by replacing the requirement for a minimum level of PEEP in patients receiving ventilation with a minimum level of airflow in patients receiving HFNO, but still using the oxygenation and chest radiographic criteria [10, 11]. A cutoff of 30 L/min for airflow was chosen, because the favorable effects on oxygenation and respiratory drive are achieved at this flow rate [5, 12]. In addition, at this airflow, HFNO may result in a level of pressure at the end of expiration of 2–5 cm H_2_O that may lead to recruitment of atelectatic distal airspaces, similar to how PEEP with ventilation may lead to lung recruitment [13, 14]. It is yet uncertain, though, if this broadened definition results in cohorts of patients receiving HFNO and patients receiving ventilation with comparable outcomes. It is also uncertain if the PaO_2_/FiO_2_ cutoffs for risk of death classification creates cohorts of HFNO patients with meaningful differences in outcomes, alike in ARDS patients receiving ventilation.

We designed a study, named ‘Practice of Adjunctive Therapies in Intensive Care Unit Patients with Coronavirus Disease 2019’ (PRoAcT–COVID), wherein we compared baseline characteristics and outcomes between patients that started with HFNO with patients that started with ventilation for acute hypoxemic failure due to coronavirus disease 2019 (COVID-19). We investigated whether a broadened definition of ARDS with use of PaO_2_/FiO_2_ cutoffs would result in distinct cohorts of HFNO patients with contrasting mortality rates, as previously shown in ARDS patients receiving ventilation.

## Methods

### Study design and participants

This is a preplanned analysis of PRoAcT–COVID [15], an investigator-initiated, nationwide, multicenter, observational study in critically ill acute hypoxemic COVID-19 patients admitted to an intensive care unit (ICU) in the first 3 months of the second wave of the national outbreak in the Netherlands. The study protocol was approved by local institutional Review Board of the Amsterdam UMC, location ‘AMC’. Due to the observational nature of this study, the need for patient informed consent was waived. The study protocol was pre-published [15, 16]. The study was registered at ClinicalTrials.gov (study identifier NCT04719182). The statistical analysis plan of the study was finalized before cleaning and closing of the database and can be found in the supplemental material.

Patients were eligible for participation in PRoAcT–COVID if (i) ≥ 18 years of age; and (ii) admitted to one of the participating ICUs from October 2020 through January 2021; (iii) for COVID-19 that was confirmed by reverse transcriptase–polymerase chain reaction (RT–PCR). At the time of conduct of PRoAcT–COVID, in the Netherlands all patients that needed escalation of oxygen therapy or respiratory care were promptly admitted to an ICU—this means that neither HFNO, nor NIV and invasive ventilation were started before admission to an ICU in nearly all patients. PRoAcT–COVID itself had no exclusion criteria. For this preplanned analysis, we excluded patients that did not start with HFNO or ventilation shortly after arrival in the ICU, and patients that did not have a PaO_2_/FiO_2_ < 300 mmHg after start of HFNO or ventilation. We also excluded patients that were transferred under or started with extracorporeal life support within the first hours after arrival in the ICU.

Patients were included in the HFNO group when they started with HFNO on ICU admission, and HFNO was continued to the next calendar day or longer. Patients were included in the ventilation group if they started with ventilation on ICU admission.

### Data collection

The following baseline and demographic variables were collected—sex, age, weight and height, home medication and comorbidities, first day with symptoms, day of definite diagnosis of COVID-19, date of hospital admission and date of ICU admission. Disease severity scores, including the Simplified Acute Physiology Score (SAPS) II on ICU admission, and daily Sequential Organ Failure Assessment (SOFA) scores were also collected. We captured blood gas analyses results, and the following respiratory variables on the first calendar day of ICU stay—PEEP and fraction of inspired oxygen (FiO_2_) in patients receiving ventilation, and airflow and FiO_2_ in patients receiving HFNO. On day 90 we collected the last day in the ICU and hospital, and life status at ICU and hospital discharge. A patient was considered as free from respiratory support if alive and weaned from NIV, invasive ventilation, and HFNO.

### Definitions

Patients receiving HFNO were classified as having ARDS when fulfilling all criteria as in the original Berlin definition, wherein the minimum level of PEEP was replaced with a minimum level of airflow [10]*.* Patients receiving ventilation had to fulfil all criteria as in the original Berlin definition. To be included in the ventilation group patients exclusively received invasive ventilation. Patients receiving other forms of respiratory support, e.g., CPAP or NIV were excluded from the current analysis.

ARDS patients receiving ventilation were classified as having mild, moderate or severe arterial hypoxemia using the PaO_2_/FiO_2_ cutoffs as of the original Berlin definition [1]. ARDS patients receiving HFNO were classified using the PaO_2_/FiO_2_ cutoffs as of the original Berlin definition, and using PaO_2_/FiO_2_ tertiles.

### Outcomes

The primary endpoint of this analysis was ICU mortality, defined as death before ICU discharge. Secondary endpoints were hospital mortality; mortality on days 28 and 90; need for ventilation within 7 days in patients that started with HFNO; the number of days free from HFNO or ventilation, using a definition as reported before [17]; and ICU and hospital length of stay.

### Power calculation

We did not perform a power calculation. The number of available patients in the database of PRoAcT–COVID served as the sample size.

### Statistical analysis

Descriptive statistics were used to report baseline characteristics, respiratory support characteristics and outcomes. Categorical variables were reported as numbers and their relative proportions, continuous variables were reported as medians (quartile 25%–quartile 75%). Comparisons between groups were made using Fisher’s exact tests for categorical variables, and Wilcoxon rank-sum tests for continuous variables.

First, hazard ratios (HRs) were calculated for ICU-mortality in patients receiving HFNO and ICU-mortality in patients receiving ventilation using a shared frailty model with center as frailty. The following baseline variables were added to the model as covariates: age, sex, BMI, PaO_2_/FiO_2_, creatinine, fluid balance, hypertension, heart failure, diabetes, COPD and malignancy. These baseline variables were selected to clinical relevance and as used in previous studies [18].

For the first day in ICU, the PaO_2_/FiO_2_ was calculated using data collected 1 h after start of respiratory support, i.e., HFNO or ventilation; for the following days, it was calculated using data collected at a fixed timepoint in the morning. Thereafter, all patients were classified as having mild, moderate or severe arterial hypoxemia, using the PaO_2_/FiO_2_ as used in the original Berlin definition. HR for ICU-mortality was calculated for these groups. Then, HFNO patients were classified having mild, moderate or severe hypoxemia, using tertiles of the PaO_2_/FiO_2_, and HR for ICU-mortality was calculated. A shared frailty model with center as frailty was used to calculate HRs.

Length of ICU stay, hospital stay and duration of respiratory support were compared between patients receiving HFNO and patients receiving ventilation using competing risk analyses with ICU-mortality, hospital mortality and mortality before cessation of respiratory support, respectively, as the competing risk.

All analyses were conducted in R v.4.0.2 (R Foundation for Statistical Computing, Vienna, Austria. URL https://www.R-project.org/) and *p* < 0.05 was considered statistically significant. As the analyses on secondary endpoints were considered exploratory, no correction for multiple testing was performed.

## Results

**Fig. 1:**
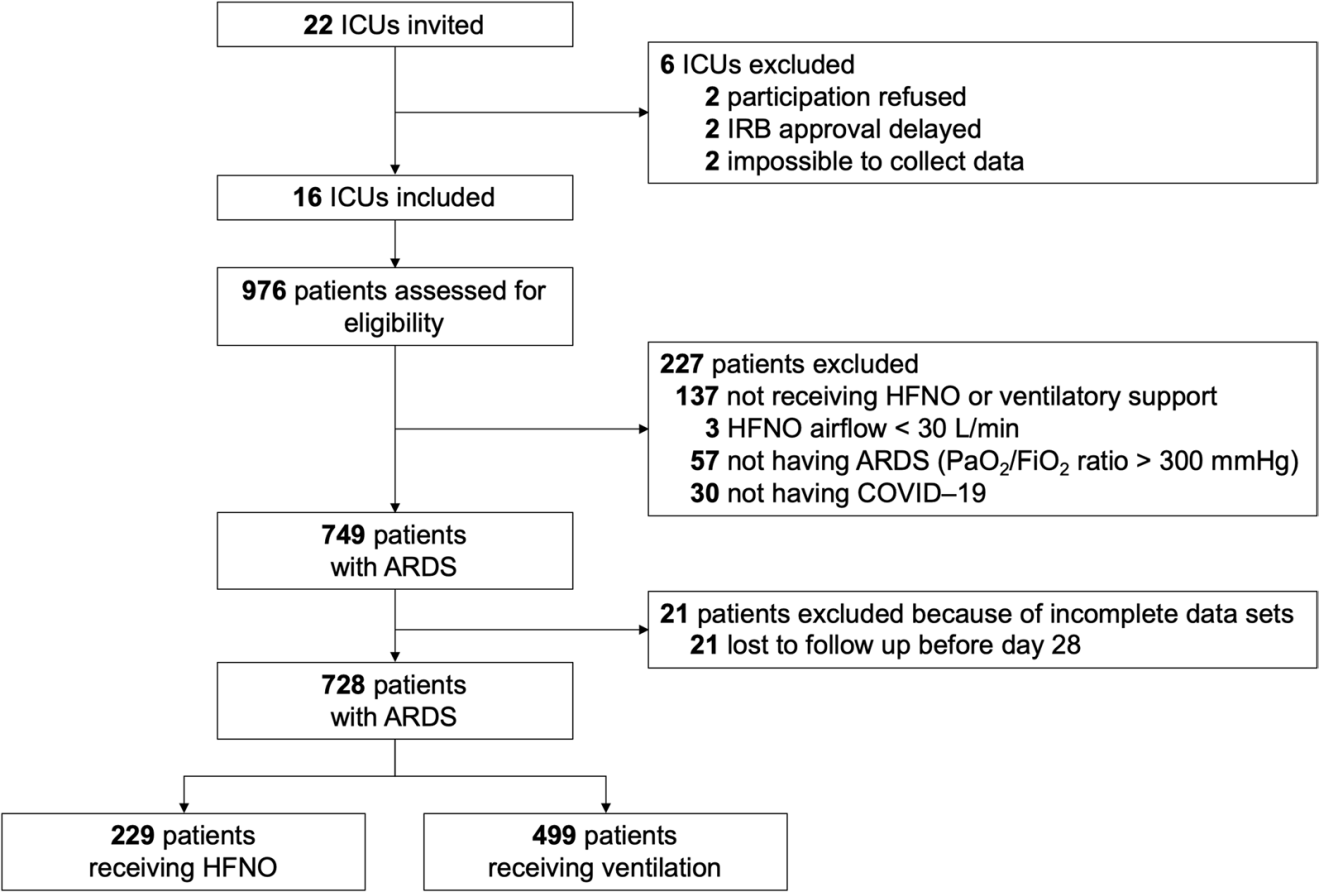
CONSORT flowchart of patients included in this analysis. *ARDS* acute respiratory distress syndrome, *FiO*_*2*_ fraction of inspired oxygen, *HFNO* high-flow nasal oxygen, *ICU* intensive care unit, *IRB* institutional review board, *PaO*_*2*_ partial pressure of oxygen

Between September 1, 2020, and January 1, 2021, 976 patients were screened in a total of 16 ICUs (Fig. 1). The single reason for exclusion from PRoAcT–COVID was having acute hypoxemic respiratory failure that was not due to COVID-19. Two main reasons for exclusion from the current analysis were having received CPAP or NIV on the first calendar day in the ICU, and not having a sufficiently low PaO_2_/FiO_2_ to be classified as having ARDS. Of the remaining 728 patients, 229 started with HFNO and 499 started with invasive ventilation. Most patients were male and having a medical history of arterial hypertension and diabetes (Table 1 and Additional file 1: Tables S1–S3). All patients met the radiologic criteria for ARDS as in the original Berlin definition of ARDS. Patients receiving HFNO had a lower median SAPS II and median SOFA score than ventilated patients. Characteristics of respiratory support are presented in Additional file 1: Figure S1. Of all HFNO patients, 105 continued with ventilation at a later time point, i.e. after the second calendar day in the ICU.

**Table 1:** Demographics, respiratory support characteristics, and hypoxemia severity

	HFNO *N* = 229	Ventilation *N* = 499	*p*
Demographics			
Age, years (median [IQR])	66 [60–73]	67 [59–73]	0.634
Male gender, N (%)	172 (75.1)	368 (73.7)	0.765
Height, cm (median [IQR])	175 [168–180]	174 [168–180]	0.624
Weight, kg (median [IQR])	85 [76–95]	87 [78–100]	0.063
BMI, kg/m^2^ (median [IQR])	28 [25–32]	29 [26–33]	0.042
SAPS II (median [IQR])*	(110/229) 31 [22–36]	(214/499) 39 [32–46]	< 0.001
SOFA score (median [IQR])*	(146/229) 4 [3–6]	(291/499) 6 [4–8]	< 0.001
Comorbidities, n (%), yes	199 (86.9)	426 (85.4)	0.664
Arterial hypertension	88 (38.4)	183 (36.7)	0.710
Heart failure	61 (26.6)	124 (24.8)	0.672
Diabetes mellitus	77 (33.6)	148 (29.7)	0.323
Chronic kidney disease	22 (9.6)	36 (7.2)	0.337
Liver cirrhosis	2 (0.9)	2 (0.4)	0.794
COPD	56 (24.5)	112 (22.4)	0.615
Active hematological cancer	6 (2.6)	17 (3.4)	1.000
Active solid cancer	13 (5.7)	18 (3.5)	0.219
Metastatic cancer	4 (1.7)	5 (1.0)	0.629
Neuromuscular disease	7 (3.1)	9 (1.8)	0.424
Immunosuppression	3 (1.3)	15 (3.0)	0.266
Respiratory Support Characteristics			< 0.001
PEEP, cm H_2_O (median [IQR])	N.A	12 [10–13]	
Airflow, L/min (median [IQR])	50 [50–60]	N.A	
FiO_2_ (median [IQR])	70 [60–85]	50 [40–61]	
CPAP before ICU, N (%)	0 (0.0)	1 (0.2)	
NIV before ICU, N (%)	1 (0.3)	5 (1.0)	
Hypoxemia severity			
PaO_2_/FiO_2_, mmHg (median [IQR])	92 [72–123]	150 [117–188]	< 0.001
PaO_2_/FiO_2_ ranges			< 0.001
200–300 mmHg	6 (2.6)	89 (17.8)	
100–200 mmHg	91 (39.7)	340 (68.1)	
< 100 mmHg	132 (57.6)	70 (14.0)	

*BMI* body mass index, *CPAP* continuous positive airway pressure, *COPD* chronic obstructive pulmonary disease, *FiO*_*2*_ fraction of inspired oxygen, *HFNO* high–flow nasal oxygen, *NIV* non–invasive ventilation, *PaO*_*2*_ partial pressure of arterial oxygen, *PEEP* positive end–expiratory pressure, *SAPS* simplified acute physiology score, *SOFA* sequential organ failure assessment

^*^We did not have all disease severity scores in all patients; we could only use the score that was recorded in patient record files

**Fig. 2:**
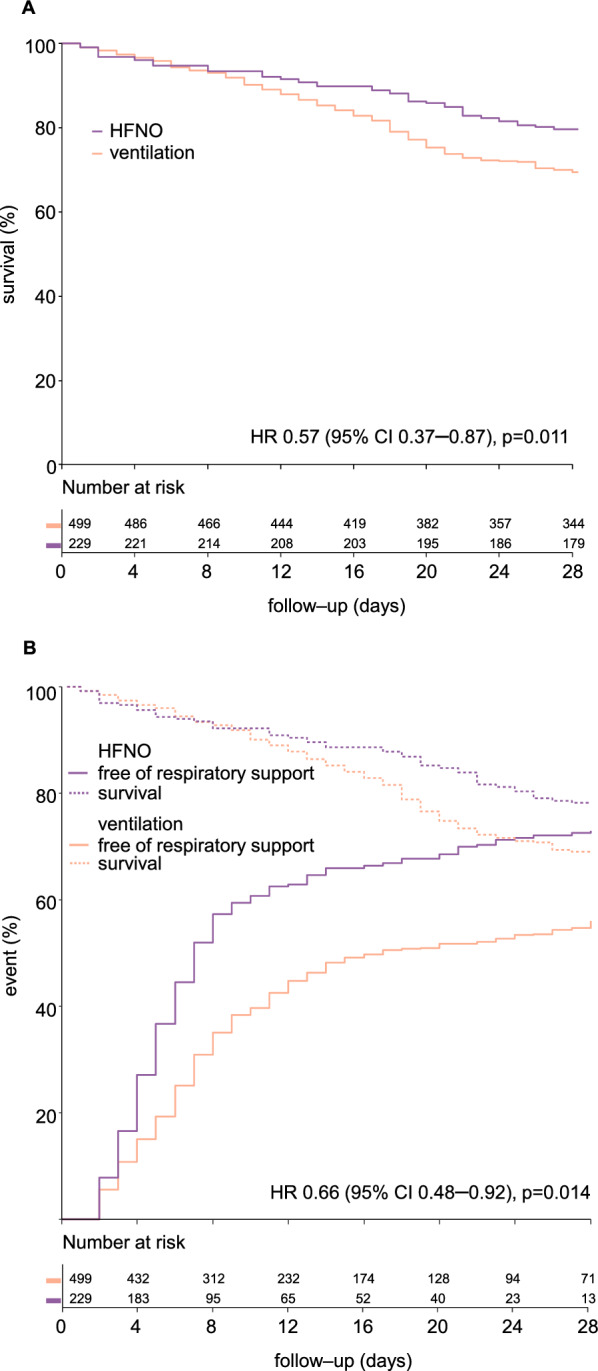
**A** 28-day survival and **B** cumulative incidence of liberation of respiratory support in patients receiving HFNO compared to patients receiving ventilation. For survival the adjusted hazard ratio is shown. **A** shows survival in the first 28 days, and **B** shows survival until the day of extubation. The cumulative incidence of liberation from respiratory support is shown as unadjusted hazard ratio with center as random effect. *CI* confidence interval, *HFNO* high-flow nasal oxygen, *HR* hazard ratio

Compared to patients receiving ventilation, HFNO patients had a lower ICU mortality (22.7 versus 35.6%; HR 0.57 [0.37–0.87], p = 0.011) (Table 2). HFNO patients also had a lower 28 and 90 days, and hospital mortality, had a longer stay in ICU and in hospital, and more respiratory support free days (Table 2 and Additional file 1: Figure S2). The cumulative incidence of liberation from respiratory support with death as a competing risk was higher in patients receiving HFNO (Fig. 2 and Additional file 1: Figure S2).

**Table 2:** Clinical endpoints

	HFNO (*N* = 229)	Ventilation (*N* = 499)	*p*
Primary endpoint			
ICU mortality, *n*/*N* (%)	52/229 (22.7)	172/483 (35.6)	0.001
Secondary endpoints			
28–day mortality, *n*/*N* (%)	50/229 (21.8)	159/499 (31.9)	0.007
90–day mortality, *n*/*N* (%)	57/229 (24.9)	178/483 (36.9)	0.002
Hospital mortality, *n*/*N* (%)	56/229 (24.5)	178/483 (36.9)	0.001
ICU length of stay, days (median [IQR])*	(225/229) 8 [4–19]	(462/483) 13 [7–26]	< 0.001
ICU length of stay in survivors, days (median [IQR])*	(168/229) 7 [4–13]	(285/483) 11 [7–30]	< 0.001
Hospital length of stay, days (median [IQR])*	(224/229) 14 [10–25]	(462/483) 19 [13–31]	< 0.001
Hospital length of stay in survivors, days (median [IQR])*	(167/229) 14 [10–28]	(284/483) 23 [14–45]	< 0.001
Need for intubation and ventilation, *N* (%)	105 (45.8)		
Respiratory support free days (median [IQR])	22 [0–25]	9 [0–22]	< 0.001

Respiratory support free days is the number of days free from HFNO or positive pressure ventilation, and alive on day 28

*HFNO* high–flow nasal oxygen, *ICU* intensive care unit, *IQR* interquartile range, *n* number, *N* total number

^*^Not available in all patients

**Fig. 3:**
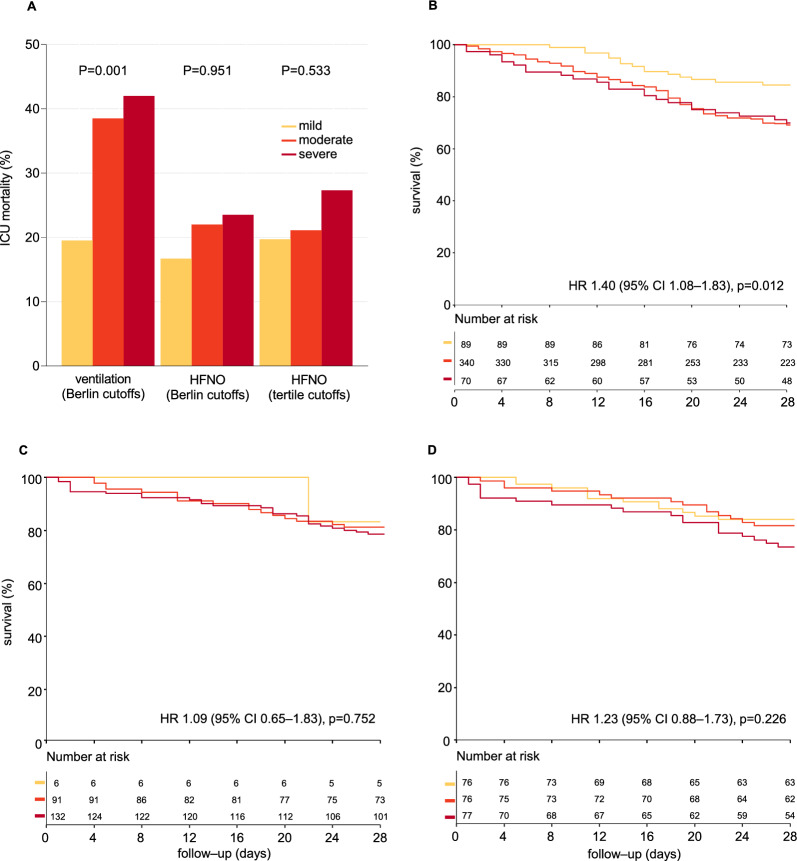
Outcome in patients receiving ventilation or HFNO, in risk of death groups based on the PaO_2_/FiO_2_ cutoffs as in the Berlin definition of ARDS, and in patients receiving HFNO, in risk of death groups based on the tertile PaO_2_/FiO_2_ cutoffs. ICU mortality is shown in (**A**). PaO_2_/FiO_2_ cutoffs as in the Berlin definition were 200–300 (mild ARDS), 100–200 (moderate ARDS) and < 100 mmHg (severe ARDS). PaO_2_/FiO_2_ cutoffs based on tertiles were 110–300 (mild hypoxemia), 80–110 (moderate hypoxemia) and < 80 mmHg (severe hypoxemia). **B** shows ICU survival for patients receiving ventilation according to the risk of death groups based on the PaO_2_/FiO_2_ cutoffs as in the Berlin definition of ARDS. **C** shows ICU survival for patients receiving HFNO according to the risk of death groups based on the PaO_2_/FiO_2_ cutoffs as in the Berlin definition of ARDS. **D** shows ICU survival for patients receiving HFNO using PaO_2_/FiO_2_ cutoffs based on tertiles. Unadjusted hazard ratios with center as random effect are shown. *CI* confidence interval, *FiO*_*2*_ fraction of inspired oxygen, *HFNO* high-flow nasal oxygen, *HR* hazard ratio, *PaO*_*2*_ partial pressure of oxygen

In ventilated patients, ICU mortality and other outcomes worsened stepwise with increasing severity of ARDS, i.e., from mild to moderate-to-severe ARDS, using the PaO_2_/FiO_2_ cutoffs for severity classification as in the original Berlin definition (Fig. 3 and Additional file 1: Figure S3). In HFNO patients, there was less difference in mortality between the severity classes based on these PaO_2_/FiO_2_ cutoffs, and most contrast was seen between patients classified as having mild ARDS versus patients with moderate-to-severe ARDS. Using PaO_2_/FiO_2_ tertiles instead of the original PaO_2_/FiO_2_ cutoffs in these patients shifted mortality contrast from between mild versus moderate-to-severe ARDS to between mild-to-moderate versus severe ARDS.

## Discussion

The findings of this study in patients with COVID-19 that needed escalation of respiratory care for acute hypoxemic respiratory failure can be summarized as follows: (i) compared to patients that started with ventilation, patients that started with HFNO had lower mortality rates; (ii) a higher number of days free from respiratory support; and (iii) a shorter length of stay in ICU and hospital. Using the cutoffs for PaO_2_/FiO_2_ as in the original Berlin definition, (iv) resulted in three cohorts of patients with differences in mortality; (v) but with insufficient contrast between patients with moderate arterial hypoxemia and patients with severe arterial hypoxemia.

Our study has several strengths. PRoAcT–COVID included a large number of critically ill hypoxemic COVID-19 patients in various types of care facilities, including academic centers, and teaching and non-teaching centers, adding to the generalizability of our findings. In the second wave of the national outbreak of COVID-19, we had a policy to immediately admit patients that needed escalation of respiratory support, including HFNO or ventilation, to an ICU, meaning that we were able to capture data on a homogenous cohort of patients. Prior to data collection of the study, all data collectors underwent extensive training to guarantee a high quality of the data captured. PRoAcT–COVID had no exclusion criteria other than having another cause for acute hypoxemic respiratory failure than COVID-19, and for this analysis, we excluded only patients that were not receiving HFNO or ventilation on the first day in the ICU. According to the broadened definition, we restricted the analysis to HFNO patients with an airflow of 30 L/min or more. Follow-up was near to complete, and the percentage of missing data was acceptable. We strictly followed the predefined and straightforward analysis plan.

The findings of our study extend the current knowledge regarding diagnosis of and risk of death classification in patients receiving HFNO for acute hypoxemic respiratory failure. One study from Italy showed that most patients receiving HFNO and classified as having ARDS still fulfil the Berlin definition of ARDS after escalation to invasive ventilation [19]. That study also showed that severity of hypoxemia changes after intubation. After start of ventilation, lower FiO_2_ could be used or PaO_2_ had increased, causing an improvement in the PaO_2_/FiO_2_. This change causes a change in the risk of death, as it is based on the level of arterial hypoxemia. A change in risk of death was also seen in a study from Sweden [20]. One study from Spain showed similar patterns of plasma biomarkers of epithelial and endothelial lung injury in patients receiving HFNO and patients receiving ventilation for acute hypoxemic respiratory failure [7]. Different from these three studies, we compared patients that started with HFNO with patients that started with ventilation that were classified as having ARDS, replacing the PEEP level for airflow on the first day in ICU. This allowed us to come to a more practical comparison, one that is closer to a real-life scenario in which decisions regarding therapy, probably influenced by the diagnosis ARDS, and risk classifications need to be made early after ICU admission.

We show that risk of death classification based on PaO_2_/FiO_2_ results in three patient cohorts with increasing mortality rates. There was little contrast between patients with moderate arterial hypoxemia and patients with severe arterial hypoxemia, however, and this classification also led to a small group of patients in the mild arterial hypoxemia group. At the moment of data collection, it was unclear whether titration of HFNO settings followed a protocol, and also whether weaning from HFNO was protocolized. In the absence of a protocol for titration of HFNO settings, healthcare workers may have favored the use of (too) high FiO_2_ and airflow settings, even when patients improved. Obviously, this affects patient classification when using PaO_2_/FiO_2_. Indeed, a too liberally set FiO_2_ may result in an erroneously low PaO_2_/FiO_2_, thereby overestimating the severity of arterial hypoxemia. In addition, we are uncertain whether blood sampling for gas analysis was performed when a patient was having his or her mouth shut. When a patients’ mouth would have been open, there could have been admixture with room air. The benefit of creating some PEEP with HFNO may also disappear when patients are having their mouth open [14]. Finally, we should hold in mind the non-linear relationship between FiO_2_ and the PaO_2_/FiO_2_ [21, 22].

HFNO has become an attractive alternative for ventilation in patients with acute hypoxemic respiratory failure, at least in certain patients [3, 23]. During the ongoing COVID-19 pandemic, we witnessed a further increase in its use in the Netherlands [18, 24]. The sharp increase in HFNO use in COVID-19 patients may have been driven by the characteristics of the disease, wherein many patients suffer more from severe arterial hypoxemia than from impaired respiratory mechanics [25]. It is very well possible that these patients disappeared from the cohorts published upon at later timepoints in the pandemic [26]. It may also explain the differences with other investigations [19, 20], but confirms that patients that can be treated with HFNO have better outcomes than patients that receive or escalate to ventilation.

We attempted to improve risk classification by using cutoffs for PaO_2_/FiO_2_ based on tertiles. This increased the number of patients with mild arterial hypoxemia. However, contrast between patients with mild and moderate arterial hypoxemia became less. It remains uncertain whether risk classification for death in patients receiving HFNO should be based only on PaO_2_/FiO_2_. Another factor to consider is the level of airflow. Several studies have shown that the amount of positive pressure increases substantially from 40 L/min to 60 L/min airflow [14, 27]. Thus, the airflow may have an effect on the severity of arterial hypoxemia, and as such may also be useful in risk for death classification [28, 29].

One important challenge of this study is the interpretation of the comparability of the two groups. While it seems that ventilated patients were sicker than patients receiving HFNO seen the differences in the disease severity scores, paradoxically hypoxemic failure appeared to be more severe in patients receiving HFNO, i.e., they had a lower PaO_2_/FiO_2_. These differences can be explained by the fact that ventilation per se is part of the disease severity score, meaning that ventilated patients per definition received more severity points than patients receiving HFNO. Also, ventilated patients received high levels of PEEP, which improves oxygenation and, therefore, influences the PaO_2_/FiO_2_. However, our findings do show that early mortality between the two groups is similar and therefore it appears the two groups are comparable. Furthermore, this study is a representation of what happened in the second wave of the COVID-19 pandemic, during which an understandable emphasis was placed on patient care rather than on administration—this for instance means that in some patients not all disease severity scores were calculated and reported. However, in all patients at least one severity score could be collected.

Other limitations included the following. Due to the observational nature of this study the findings of the current analysis should be seen as exploratory and can only provide a rationale for further investigations. In addition, in the Netherlands CPAP or NIV were seldom used in COVID-19 patients. If these forms of respiratory support were used, which mainly happened outside of the ICU, it was given as part of palliative care. This limits the generalizability of the findings of our study to countries or regions where CPAP or NIV are used more often, or where it is not used as part of palliative care. The findings of our study reflect the applicability of the broadened ARDS definition only in patients with COVID-19. It remains unclear whether the findings of our study can be generalized to patients receiving HFNO for acute hypoxemic respiratory failure due to other causes. In addition, none of the patients in the ventilated group started with NIV, limiting the generalizability of our study to settings where NIV is used more often. Data regarding do–not–intubate orders could not be reliably collected in PRoAcT–COVID. These orders could have limited life sustaining treatments and thereby could have interfered with the study results. Also, we should consider that the unprecedented demand on the ICUs and consequently resource limitations may have influenced clinical decision making. Finally, even if this is the largest study to date on the usefulness of a broadened Berlin definition, we remain underpowered to show a statistically significant difference between the patients with worsening arterial hypoxemia.

In conclusion, the use of a broadened Berlin definition of ARDS allows an earlier diagnosis in patients that start with HFNO. However, HFNO patients that meet the broadened definition have a lower mortality than patients receiving ventilation. Further refinement of the broadened definition, including cutoffs for the severity of ARDS, remains needed.

## Supplementary Information

**Additional file 1: Table S1.** Demographics mild ARDS according to Berlin definition. **Table S2.** Demographics moderate ARDS according to Berlin definition. **Table S3.** Demographics severe ARDS according to Berlin definition. **Figure S1.** Cumulative frequency distribution of PaO2/FiO2 ratio, FiO2, and PEEP or flow per severity class in HFNO and ventilation. **Figure S2.** Cumulative incidence of ICU discharge (**A**) and hospital discharge (**B**) in HFNO compared to ventilation. **Figure S3.** Hospital (**A**), 28–day mortality (**B**) and all–cause 90–day mortality (**C**) per severity class.

## Data Availability

A de-identified dataset will be made available upon request to the corresponding authors 1 year after the publication of this study. The request must include a statistical analysis plan.
