# Supplementary material for: Broadening the Berlin definition of ARDS to patients receiving high-flow nasal oxygen: an observational study in patients with acute hypoxemic respiratory failure due to COVID-19
Source: Ann Intensive Care. 2023 Jul 14;13:64. doi: 10.1186/s13613-023-01161-6 (PMC10349031; doi:10.1186/s13613-023-01161-6)
Supplement: Supplementary file 1 — Additional file 1: Table S1. Demographics mild ARDS according to Berlin definition. Table S2. Demographics moderate ARDS according to Berlin definition. Table S3. Demographics severe ARDS according to Berlin definition. Figure S1. Cumulative frequency distribution of PaO2/FiO2 ratio, FiO2, and PEEP or flow per severity class in HFNO and ventilation. Figure S2. Cumulative incidence of ICU discharge (A) and hospital discharge (B) in HFNO compared to ventilation. Figure S3. Hospital (A), 28–day mortality (B) and all–cause 90–day mortality (C) per severity class. [file 13613_2023_1161_MOESM1_ESM.docx]

SUPPLEMENT TO

**Broadening the Berlin Definition of ARDS to Patients Receiving High–flow Nasal Oxygen––an observational study in patients with acute hypoxemic respiratory failure due to COVID–19**

Fleur–Stefanie L.I.M. van der Ven, MD^1,7†^; Christel M.A. Valk, MD, PhD^1†^; Siebe Blok, MD^1†^; Michelle G. Brouwer, MD^1^; Dai Ming Go, BSc^1^; Amanda Lokhorst, BSc^1^; Pien Swart, BSc^1^; David M.P. van Meenen, MD, PhD^1,2^; Frederique Paulus, PhD^1,4^, Marcus J. Schultz, MD, PhD^1,3,5,6^ for the PRoAcT–COVID* study investigators**

^†^contributed equally

*‘Practice of Adjunctive Therapies in COVID–19 Patients’

**Correspondence:**

Fleur–Stefanie L.I.M. van der Ven

Department of Intensive Care

Amsterdam University Medical Centers, location ‘AMC’

Amsterdam

The Netherlands

E–mail: [f.ven@amsterdamumc.nl](mailto:f.ven@amsterdamumc.nl)

**CONTENT**

**eTable and eFigures**

eTable 1: Demographics mild ARDS according to Berlin definition page 3

eTable 2: Demographics moderate ARDS according to Berlin definition page 4

eTable 3: Demographics severe ARDS according to Berlin definition page 5

eFigure 1: Cumulative frequency distribution of PaO_2_/FiO_2_ ratio, FiO_2_, and

PEEP or flow per severity class in HFNO and ventilation page 6

eFigure 2: Cumulative incidence of ICU discharge (A) and hospital discharge (B)

in HFNO compared to ventilation page 7

eFigure 3: Hospital (A), 28–day mortality (B) and all–cause 90–day mortality (C)

per severity class page 8

**Statistical Analysis Plan**

Statistical analysis plan page 9

| **eTable 1. Demographics mild ARDS according to the Berlin definition** | | |
| --- | --- | --- |
|  | **HFNO** | **ventilation** |
|  | **(N = 6)** | **(N = 89)** |
| age, years (median [IQR]) | 58 [53–65] | 65 [56–72] |
| male gender, N (%) | 6 (100.0) | 65 (73.0) |
| height, cm (median [IQR]) | 178 [176–180] | 175 [167–180] |
| weight, kg (median [IQR]) | 85 [79–93] | 85 [78–95] |
| BMI, kg/m^2^ (median [IQR]) | 28 [26–28] | 29 [26–32] |
| SAPS II (median [IQR])* | 24 [16–36] | 37 [31–48] |
| SOFA score (median [IQR])* | 3 [–3] | 5 [4–7] |
| comorbidities, n (%), yes | 6 (100.0) | 78 (87.6) |
| arterial hypertension | 4 (66.7) | 33 (37.1) |
| heart failure | 1 (16.7) | 4 (4.5) |
| diabetes mellitus | 2 (33.3) | 28 (31.5) |
| chronic kidney disease | 1 (16.7) | 6 (6.7) |
| liver cirrhosis | 0 (0) | 0 (0) |
| COPD | 0 (0) | 4 (4.5) |
| active hematological cancer | 0 (0) | 2 (2.2) |
| active solid cancer | 0 (0) | 2 (2.2) |
| metastatic cancer | 0 (0) | 1 (1.1) |
| neuromuscular disease | 0 (0) | 3 (3.4) |
| immunosuppression | 0 (0) | 3 (3.4) |
| abbreviations: BMI = body mass index; CPAP = continuous positive airway pressure; COPD = chronic obstructive pulmonary disease; FiO_2_ = fraction of inspired oxygen; HFNO = high–flow nasal oxygen; NIV = non–invasive ventilation; PaO_2_ = partial pressure of arterial oxygen; PEEP = positive end–expiratory pressure; SAPS = simplified acute physiology score; SOFA = sequential organ failure assessment. | | |

| **eTable 2. Demographics moderate ARDS according to the Berlin definition** | | |
| --- | --- | --- |
|  | **HFNO** | **ventilation** |
|  | **(N = 91)** | **(N = 340)** |
| age, years (median [IQR]) | 64 [59–73] | 67 [59–73] |
| male gender, N (%) | 70 (76.9) | 255 (75.0) |
| height, cm (median [IQR]) | 175 [170–183] | 174 [168–180] |
| weight, kg (median [IQR]) | 83 [76–94] | 88 [78–100] |
| BMI, kg/m^2^ (median [IQR]) | 27 [25–31] | 29 [26–33] |
| SAPS II (median [IQR])* | 30 [22–34] | 40 [33–46] |
| SOFA score (median [IQR])* | 4 [3–5] | 7 [5–8] |
| comorbidities, n (%), yes | 77 (84.6) | 289 (85.0) |
| arterial hypertension | 33 (36.3) | 123 (36.2) |
| heart failure | 5 (5.5) | 15 (4.4) |
| diabetes mellitus | 29 (31.9) | 101 (29.7) |
| chronic kidney disease | 12 (13.2) | 24 (7.1) |
| liver cirrhosis | 0 (0) | 2 (0.6) |
| COPD | 9 (9.9) | 34 (10.0) |
| active hematological cancer | 3 (3.3) | 10 (2.9) |
| active solid cancer | 6 (6.6) | 13 (3.8) |
| metastatic cancer | 2 (2.2) | 3 (0.9) |
| neuromuscular disease | 2 (2.2) | 6 (1.8) |
| abbreviations: BMI = body mass index; CPAP = continuous positive airway pressure; COPD = chronic obstructive pulmonary disease; FiO_2_ = fraction of inspired oxygen; HFNO = high–flow nasal oxygen; NIV = non–invasive ventilation; PaO_2_ = partial pressure of arterial oxygen; PEEP = positive end–expiratory pressure; SAPS = simplified acute physiology score; SOFA = sequential organ failure assessment. | | |

| **eTable 3. Demographics severe ARDS according to the Berlin definition** | | |
| --- | --- | --- |
|  | **HFNO** | **ventilation** |
|  | **(N = 132)** | **(N = 70)** |
| age, years (median [IQR]) | 67 [60–74] | 66.5 [58–73] |
| male gender, N (%) | 96 (72.7) | 48 (68.6) |
| height, cm (median [IQR]) | 173 [167–180] | 173 [167–178] |
| weight, kg (median [IQR]) | 85 [76–98] | 87 [80–99] |
| BMI, kg/m^2^ (median [IQR]) | 28 [25–32] | 30 [26–33] |
| SAPS II (median [IQR])* | 32 [24–37] | 34 [30–45] |
| SOFA score (median [IQR])* | 4 [3–6] | 5 [4–7] |
| comorbidities, n (%), yes | 116 (87.9) | 59 (84.3) |
| arterial hypertension | 51 (38.6) | 27 (38.6) |
| heart failure | 4 (3.0) | 4 (5.7) |
| diabetes mellitus | 46 (34.8) | 19 (27.1) |
| chronic kidney disease | 9 (6.8) | 6 (8.6) |
| liver cirrhosis | 2 (1.5) | 0 (0) |
| COPD | 24 (18.2) | 3 (4.3) |
| active hematological cancer | 3 (2.3) | 2 (2.9) |
| active solid cancer | 7 (5.3) | 2 (2.9) |
| metastatic cancer | 1 (0.8) | 1 (1.4) |
| neuromuscular disease | 5 (3.8) | 0 (0) |
| immunosuppression | 1 (0.8) | 1 (1.4) |
| abbreviations: BMI = body mass index; CPAP = continuous positive airway pressure; COPD = chronic obstructive pulmonary disease; FiO_2_ = fraction of inspired oxygen; HFNO = high–flow nasal oxygen; NIV = non–invasive ventilation; PaO_2_ = partial pressure of arterial oxygen; PEEP = positive end–expiratory pressure; SAPS = simplified acute physiology score; SOFA = sequential organ failure assessment. | | |

**eFigure 1: Cumulative frequency distribution of PaO_2_/FiO_2_ ratio, FiO_2_, and PEEP or flow per severity class in HFNO and ventilation**

abbreviations: FiO_2_ = fraction of inspired oxygen; HFNO = high–flow nasal oxygen; PaO_2_ = partial arterial oxygen pressure; PEEP = positive end–expiratory pressure.


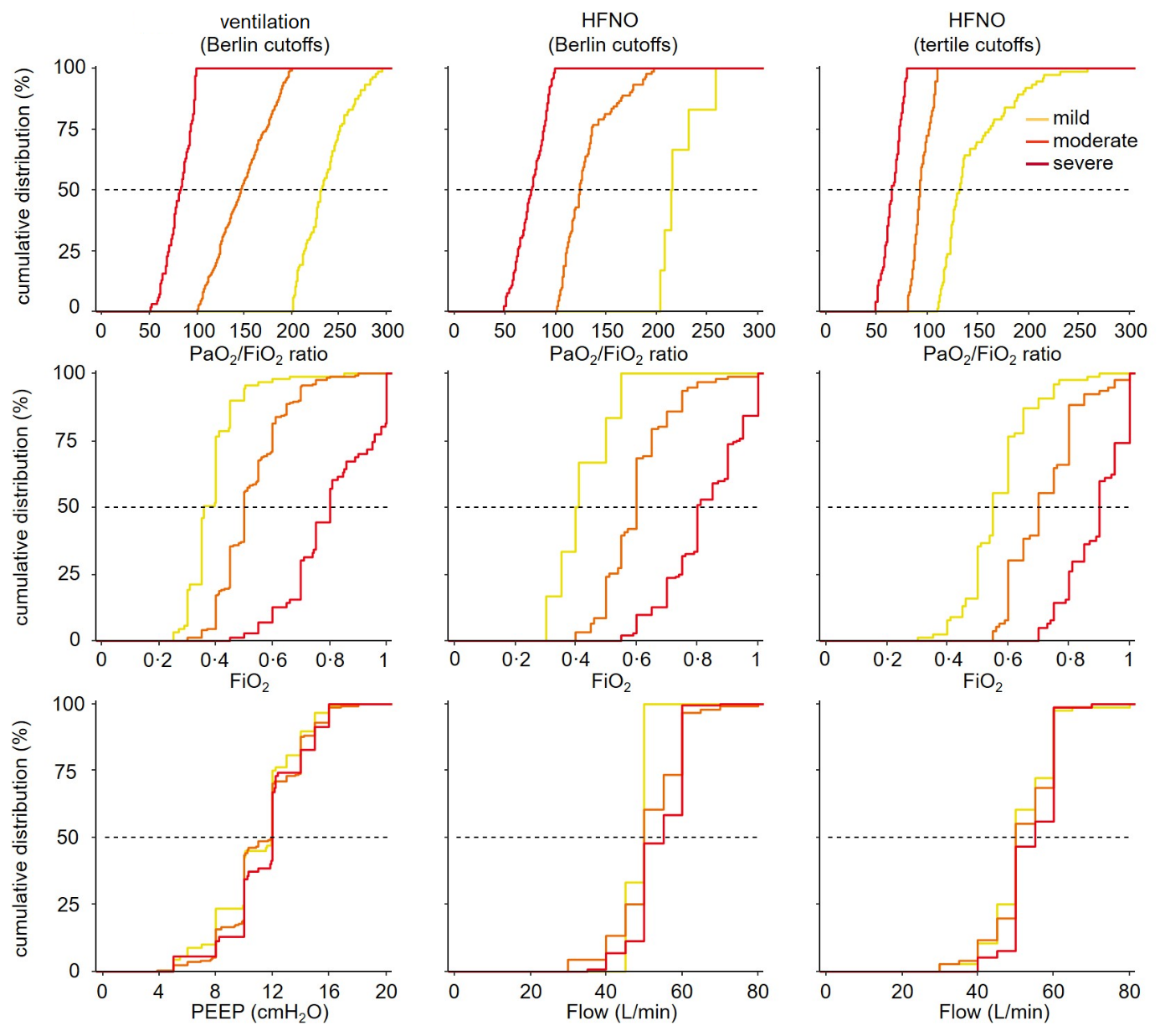


**eFigure 2: Cumulative incidence of ICU discharge (A) and hospital discharge (B) in HFNO compared to ventilation**

Unadjusted hazard–ratios with centre as random effect are shown.

abbreviations: CI = confidence interval; HFNO = high–flow nasal oxygen; HR = hazard ratio; ICU = intensive care unit.

**
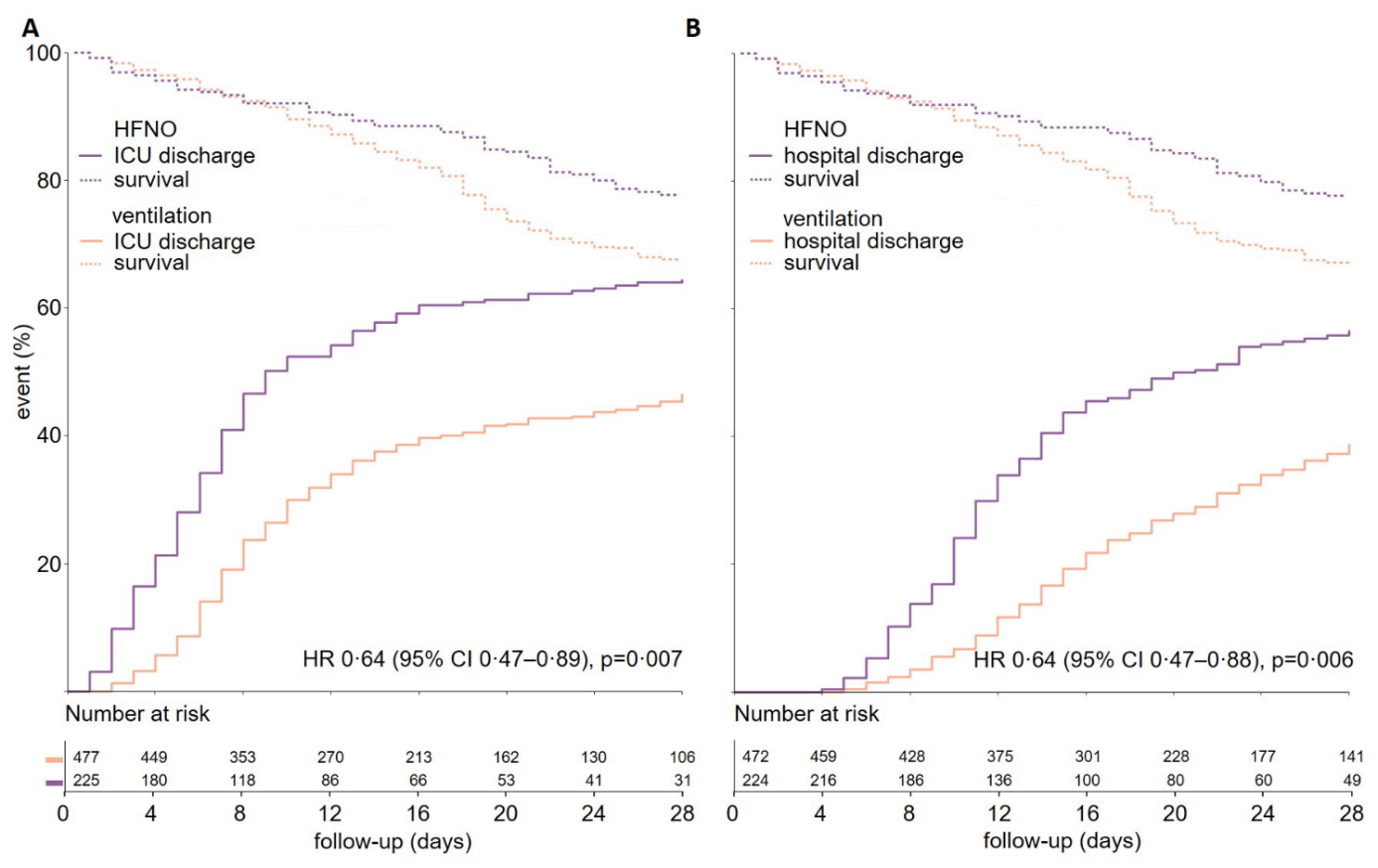
**

**eFigure 3: Hospital (A), 28–day mortality (B) and all–cause 90–day mortality (C) per severity class**

abbreviations: HFNO = high–flow nasal oxygen.


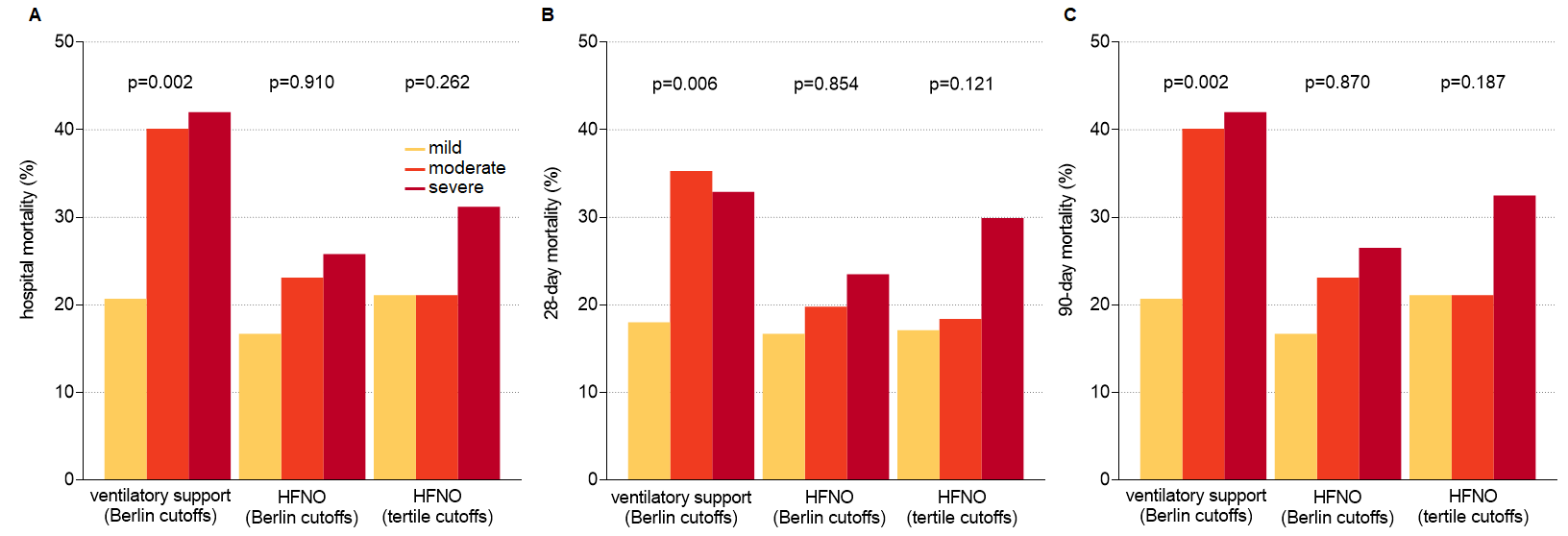


STATISTICAL ANALYSIS PLAN OF

**Broadening the Berlin Definition of ARDS to Patients Receiving High–flow Nasal Oxygen––an observational study in patients with acute hypoxemic respiratory failure due to COVID–19**

**Rationale**

Acute hypoxemic respiratory failure is a hallmark feature in patients with coronavirus disease 2019 (COVID–19) pneumonia. High–flow nasal oxygen (HFNO) is increasingly used in these patients.^1-3^ With HFNO, patients receive ventilatory support without PEEP. As the current Berlin definition for acute respiratory distress syndrome (ARDS) requires a minimal level of 5 cm H_2_O positive end–expiratory pressure (PEEP),^4^ ARDS cannot be diagnosed in these patients while it can be assumed that they have comparable pathology.

It has been proposed to use a broadened Berlin definition in acute hypoxemic respiratory patients under HFNO.^5^ It is uncertain, though, whether the use of an adjusted definition, ignoring the requirement of a minimal level of PEEP in patients under HFNO, results in comparable patient populations as when using the full definition, requiring a minimal level of PEEP in patients under ventilation. It is also uncertain whether use of the PaO_2_/FiO_2_ cutoffs as proposed in the Berlin definition lead to comparable risk groups for death.

We aim to investigate the use of a broadened definition for ARDS in COVID–19 patients under HFNO, and compare baseline characteristics and outcome in patients classified as having ARDS receiving HFNO, and in patients receiving ventilation. We also compare death classification based on PaO_2_/FiO_2_ cutoffs.

**Objectives**

1. To compare baseline characteristics and outcomes in patients under HFNO and in patients receiving ventilation, classified as having ARDS by using the Berlin definition in patients with acute hypoxemic respiratory failure due to COVID–19.
2. To compare outcomes in severity groups based on PaO_2_/FiO_2_ cutoffs in patients with acute hypoxemic respiratory failure due to COVID–19 receiving HFNO versus receiving ventilation.

**Hypotheses**

1. Using the Berlin definition for ARDS in patients with acute hypoxemic respiratory failure due to COVID–19 receiving HFNO versus receiving ventilation results in comparable patient groups with respect to baseline characteristics and outcomes.
2. Use of PaO_2_/FiO_2_ cutoffs for risk for death classification in patients with acute hypoxemic respiratory failure due to COVID–19 receiving HFNO results in cohorts of patients with contrast in mortality rates, alike in patients receiving ventilation.

**Study design**

This is a secondary analysis of the ‘Practice of Adjunctive Therapies in COVID–19 Patients’ (PRoAcT−COVID) study, a national, multicenter observational study in critically ill COVID–19 patients in the first 3 months of the second wave of the national outbreak in the Netherlands.

**Study population**

Patients were eligible for participation in PRoAcT–COVID if (1) ≥ 18 years of age; and (2) admitted to one of the participating ICUs from October 2020 through January 2021; (3) for COVID–19 that was confirmed by reverse transcriptase–polymerase chain reaction (RT–PCR).

PRoAcT–COVID itself had no exclusion criteria. For this preplanned analysis we excluded patients that did not start with HFNO or ventilation shortly after arrival in the ICU, and patients that did not have a PaO_2_/FiO_2_ < 300 mmHg after start of HFNO or ventilation. We also excluded patients that were transferred under or started with extracorporeal life support within the first hours after arrival in the ICU.

**Sample size calculation**

Due to the exploratory purpose of this study, no sample size calculation is performed; the number of patients available for this analysis serves as the sample size.

**Data collection**

For PRoAcT–COVID the following baseline and demographic variables were collected—sex, age, weight and height, home medication and comorbidities, first day with symptoms, day of a definite diagnosis of COVID–19, day of hospital admission, and day of ICU admission. In addition, disease severity score, including the Simplified Acute Physiology Score (SAPS) II and the Sequential Organ Failure Scores (SOFA) were collected during the first 24 hours in the ICU.

For this secondary analysis we will use (1) for patients receiving HFNO, the fraction of oxygen (FiO_2_) and flow rate setting, next to the arterial oxygen pressure (PaO_2_); (2) for ventilated patients, the FiO_2_, PEEP and PaO_2_.

### **Study endpoints**

The primary endpoint of this analysis was ICU mortality, defined as death before ICU discharge. Secondary endpoints were hospital mortality, and mortality on day 28 and 90; need for ventilation within seven days after in patients that started with HFNO; the number of days free from HFNO, NIV or ventilation and alive at day 28, using a definition as reported before;^7^ and ICU– and hospital length of stay.

**Statistical analysis**

Descriptive statistics will be reported as number and relative proportions for categorical variables and median (quartile 25% – quartile 75%) for continuous variables. For baseline characteristics, the HFNO group and the invasively ventilated group will be compared using Fisher exact tests for categorical variables and Wilcoxon rank–sum test for continuous variables.

First, the HFNO group will be compared with the ventilated group using survival analyses. Thereafter, all patients will be classified as mild, moderate or severe ARDS according to the Berlin definition, broadened Berlin definition and using tertiles in HFNO. Outcomes will then be compared between death classification groups.

Hazard ratios (HRs) will be calculated for 28–day mortality using an adjusted Cox proportional hazard model with center as frailty, the following variables will be considered for the final model: age, sex, BMI, PaO_2_/FiO_2_, creatinine, fluid balance, hypertension, heart failure, diabetes, COPD, and malignancy. These baseline variables are selected to clinical relevance and as used in previous studies^6^. A competing risk analysis of liberation of respiratory support, using a definition as reported before^7^, with death as a competing risk will be carried out. In addition, competing risk analyses with ICU discharge and hospital discharge with death as a competing risk will be carried out. Survival analyses, with ICU mortality as the primary endpoint, for the groups based on the classical Berlin definition, the broadened Berlin definition, and the tertiles based on PaO_2_/FiO_2_ ratio will be carried out, using a shared frailty Cox proportional hazards model with center as frailty. The same analyses will be performed for hospital and 28-day mortality as secondary outcomes.

All analyses were will be conducted in R v.4.0.2 and a p value < 0.05 will be considered statistically significant.

**References**

1. Frat JP, Thille AW, Mercat A, et al. High-flow oxygen through nasal cannula in acute hypoxemic respiratory failure. *N Engl J Med*. Jun 4 2015;372(23):2185-96. doi:10.1056/NEJMoa1503326

2. Demoule A, Vieillard Baron A, Darmon M, et al. High-Flow Nasal Cannula in Critically III Patients with Severe COVID-19. *Am J Respir Crit Care Med*. Oct 1 2020;202(7):1039-1042. doi:10.1164/rccm.202005-2007LE

3. Berlin DA, Gulick RM, Martinez FJ. Severe Covid-19. *N Engl J Med*. Dec 17 2020;383(25):2451-2460. doi:10.1056/NEJMcp2009575

4. Ranieri VM, Rubenfeld GD, Thompson BT, et al. Acute respiratory distress syndrome: the Berlin Definition. *Jama*. Jun 20 2012;307(23):2526-33. doi:10.1001/jama.2012.5669

5. Murray JF, Matthay MA, Luce JM, Flick MR. An expanded definition of the adult respiratory distress syndrome. *Am Rev Respir Dis*. Sep 1988;138(3):720-3. doi:10.1164/ajrccm/138.3.720

6. Botta M, Tsonas AM, Pillay J, et al. Ventilation management and clinical outcomes in invasively ventilated patients with COVID-19 (PRoVENT-COVID): a national, multicentre, observational cohort study. *Lancet Respir Med*. Feb 2021;9(2):139-148. doi:10.1016/s2213-2600(20)30459-8

7. Ranieri VM, Tonetti T, Navalesi P, et al. High-Flow Nasal Oxygen for Severe Hypoxemia: Oxygenation Response and Outcome in Patients with COVID-19. *Am J Respir Crit Care Med*. Feb 15 2022;205(4):431-439. doi:10.1164/rccm.202109-2163OC
